# Colostomy

**Published:** 1907-11-09

**Authors:** 


### COLOSTOMY.

(*Continued from page* 116.)

In the last two articles of this series the indications for performing left inguinal colostomy and its technique were dealt with at length, because, owing to the unfortunate frequency of carcinoma of the rectum and of the sigmoid, it is an operation which any medical man may be called upon to perform at comparatively short notice. But it need hardly be explained that if the obstruction is situated at some higher point in the large intestine other measures will be found necessary. The great difficulty which faces the surgeon is that of diagnosing the exact site of the obstruction. The main diagnostic points were dealt with in an article which appeared in The Hospital of August 17 of this year. It may be possible to diagnose the presence of a malignant stricture of the splenic flexure on the presence of one or more of the following clinical features: (1) The presence of a tumour in the left hypochondrium due either to the malignant mass itself, or, more commonly, to the fæcal material which has accumulated behind the stricture; (2) pain constantly referred to the region of the splenic flexure; (3) the presence of visible waves of peristalsis, travelling transversely across the upper abdomen from right to left and coming to an end in the left subcostal area; (4) distension of the right, but not of the left, flank; (5) a resonant note on percussion to the right of the vertebral column between the last rib and the crest of the ilium —this last is due to distension of tne ascending colon extending backwards from the point of obstruction. It is rare to find all these signs present at one time, but if more than two are found it is reasonable to argue that the obstruction is situated at the splenic flexure. What is the treatment indicated? The ideal procedure is, of course, to excise the growth and do an end-to-end anastomosis. This is rarely, if ever, practicable. The choice then lies between some form of lateral anastomosis, and transverse colostomy. The former is accompanied by much shock, and the patient's condition may be so bad that such an operation is not justified. In this case transverse colostomy—by which is meant making an artificial anus in the transverse colon—should be performed.

### Transverse Colostomy.

A transverse incision about two inches in length should be made over the centre of the right rectus abdominis. Its level depends upon the situation of the transverse colon, if this can be made out by percussion. If not, it is best to make the incision just above the level of the umbilicus. The anterior layer of the sheath of the rectus is also cut transversely and the rectus muscle is split in the line of its fibres. In this way a quadrate opening is given. The parietal peritoneum is opened and the transverse colon sought for with the finger. When brought into the wound it is, of course, easily recognised not only by the general appearances which characterise large intestine, but also by the fact that it has attached to it the great omentum. The succeeding steps of the operation are identical with those of inguinal colostomy. The only difference is due to the presence of the omentum. This may either be pierced by the forceps like the mesocolon, or any portion of it which is troublesome may be removed. As in inguinal colostomy, the bowel may be opened at a later date, or at the time of operation with a Paul's tube, and the forceps may be removed when a firm spur has been made, generally about the tenth day. There is no trouble owing to prolapse of the bowel after this operation. On the contrary, the transverse colon tends rather to sink down in the abdomen, and so cause the artificial anus to retract.

### Cæcostomy.

Cæcostomy is an emergency operation which is generally performed in cases of chronic intestinal obstruction which have become acute, its object being to get rid of the immediate results of the obstruction, so that when this has occurred some more radical treatment may be undertaken subsequently. There is much to be said in favour of its employment in these cases. When the obstruction has been allowed to become acute the abdomen is often so distended that it is impossible to locate the site of the obstruction at all definitely; but since such a condition is nearly always due to obstruction in the large gut, the cæcum is found to be distended, and opening it will relieve the condition.

The operation itself is moderately simple. A small incision is made in the same region as the incision for appendicitis. In a typical case the distended cæcum presents as soon as the parietal peritoneum has been opened. The presenting portion is fixed to the edges of the wound with silk stitches, which go through the serous and muscular coats of the bowel only. The cæcum is then opened and a Paul's tube is inserted. If the patient's condition is very bad this operation may be performed under local anæsthesia. It must be understood that the opening thus given is only a fæcal fistula, and in no sense an artificial anus, since no attempt is made to form a spur. In fact, owing to the arrangement of the peritoneum, this would not be possible. Since the opening is only made as a temporary measure to allow the effects of the obstruction to quiet down, this is a great advantage, because the opening is more easily closed subsequently. When the distension of the colon has diminished or disappeared, it may be possible to find some indication of the seat of the disease, such as a mass palpable through the abdominal wall; and this may then be excised, or some form of lateral anastomosis performed. If these measures are successful, the cæcostomy tends to close of its own accord. The process may, however, be hastened by dissecting up the skin edges and closing the aperture in the cæcum with Lembert sutures. Sometimes, however, it is necessary to do this more than once before the fistula finally closes.

